# Navigating double burden: Community health workers in rural Dominican Republic living and working through the epidemiologic transition

**DOI:** 10.1371/journal.pgph.0004378

**Published:** 2025-04-29

**Authors:** Eliana G. Armora Langoni, Deshira D. Wallace, Clare Barrington

**Affiliations:** Department of Health Behavior, UNC Gillings School of Global Public Health, University of North Carolina at Chapel Hill, Chapel Hill, North Carolina, United States of America; Nagasaki University, JAPAN

## Abstract

The epidemiological transition postulates that over time, the burden of infectious disease declines and is replaced with non-communicable diseases (NCD). Community health workers (CHW) work on prevention and treatment of infectious and NCD in low-resource settings in the context of health transitions. We explored CHWs’ experiences working through the epidemiologic transition in rural Dominican Republic and how the transition impacted their roles and communities. We conducted two semi-structured interviews each with eight CHWs. We analyzed interviews using narrative summaries and thematic coding. CHWs described themselves as change makers that drove health improvements, including child mortality reductions and advancements in social determinants of health. However, more than a transition from infectious disease to NCD, participants described a current double burden of both, and a resulting expansion of their roles and responsibilities. Increased workloads and poor remuneration, layered on top of gendered roles and expectations, were identified as threats to CHW sustainability, efficacy, and well-being. CHWs need additional support to continue their essential role strengthening the health profile of communities in transition.

## Introduction

Community health workers (CHW) are central actors in efforts to promote health goals in underserved populations across the globe [1]. CHWs are members of the communities they serve and play various roles including health educators, health system navigators, advocates, and providers of basic preventive health care [2]. Roles, remuneration, and training vary across countries, and within countries [2–4]. While CHWs have traditionally focused on infectious disease and maternal and child health in low-and-middle-income-countries (LMIC) [1,2], with changing health landscapes, CHWs are increasingly focusing on non-communicable diseases (NCD) as well [1].

In the Dominican Republic (DR), CHWs have been leveraged in governmental and non-governmental settings as extensions of the healthcare system in the face of a limited health workforce [5]. In the 1999 National Ordinance for Hospitals in the DR, CHWs were identified as part of the healthcare referral system, tasked with detecting and referring mothers with high-risk pregnancies and other patients to healthcare centers [6]. Since then, government-defined roles have expanded to include 1) administrative roles, such as assisting with epidemiological surveillance, 2) health education at community centers and in-home visits with each family, with emphasis on child and maternal health and infectious disease 3) referral and accompaniment of patients to healthcare services, and 4) community organization and serving as a liaison between the community and healthcare services [7]. CHW programs in the DR also include health care delivery roles such as administering vaccinations and certain basic medications, and clinic tasks such as taking temperatures [8]. CHWs in the DR have typically completed secondary education and receive formal trainings [5,9]. Information on payment is limited but a 2009 article reported that CHWs in the DR were paid an average of 900 Dominican pesos a month (equivalent to US$15), about one-third of the minimum wage for the public sector at the time [10,11]. One of the ongoing goals of the Ministry of Public Health is to recruit and train more CHWs to assist with promoting equitable access to health services [5].

Omran described the changing patterns of disease and mortality in his 1971 epidemiological transition theory. According to the theory, mortality is a fundamental factor in population dynamics. Yet these dynamics fluctuate according to epidemiological transitions, which consist of three stages: a period of high infectious diseases; a period where mortality and epidemics decline; and finally, a period defined by lower mortality and an increase in NCDs. Importantly, Omran argues that the biggest changes in these transitions are reduced infant, under-5, and maternal morbidity and mortality. Lastly, Omran posits that the epidemiological transition occurs in response to improved living conditions due to industrialization and urbanization [12]. However, the theory has been critiqued for not accounting for the role of poverty in delaying the stages described above as well as the permanence of infectious disease and nutrition inadequacy in poor populations, leading to more of a double burden of infectious and NCDs rather than a replacement [13]. Accordingly, the theory has been critiqued for its lack of generalizability across populations and oversimplification of disease metric meanings and transition patterns [14].

Omran developed additional models for countries that were following this process at different rates, including the delayed model, for which he used Chile, a Latin American country, as an example [12]. Alternatively, Frenk et al, suggested that Latin American countries follow a different model: the “polarized prolonged model” where countries like Mexico experience a double burden of both infectious and NCD for a prolonged period without a clear resolution, instead of transitions between defined phases [15]. The theory also posits a polarization, or disparity in disease profiles across countries and also between urban and rural areas, such that rural areas experience high levels of infectious disease and urban areas experience high levels of NCD [15].

Epidemiological data indicate that the Dominican Republic is following the patterns defined in the epidemiologic transition. Between 1990 and 2019, the NCD burden in the country increased by 21%, while the burden due to infectious, maternal, neonatal, and nutrition diseases decreased by 63% [16]. While each nation is unique, these trends align with patterns observed across Latin America [16]. During this time, considerable improvements in educational attainment and health insurance coverage were achieved in the DR. However, healthcare infrastructure, poverty, sanitation, and access to drinking water remain challenges [17].

Despite their central role in health promotion particularly for child and maternal health and infectious disease, there is limited understanding of how CHWs perceive the epidemiologic transition to have occurred in their communities and their experiences working through these transitions. Further, there are no studies that have qualitatively examined the lived experiences of CHWs in the Dominican Republic [4,18]. Understanding CHWs’ perspectives and experiences is critical to tailoring programs to the reality of long-term, community-based support for the dual burden of infectious disease and chronic disease care and management. CHW insights can also identify the potential policy and system changes required to optimize CHW programs. Thus, we aim to explore CHWs’ perspectives on the epidemiologic transition in rural communities in the Dominican Republic and the impact of the transition on their CHW role.

## Methods

### Study setting

We conducted this qualitative study from June 1 to August 31, 2015 at two community health clinics that serve over 30 rural communities in the Cibao Valley of the Dominican Republic. The Cibao Valley is an agricultural region with high production of rice, coffee, and cacao. We collaborated with Chronic Care International (CCI), a US-based non-profit that established a diabetes and hypertension program in 2010 in collaboration with the Institute for Latin American Concern (ILAC), a Dominican-based non-governmental health and development organization. ILAC has been active in the study region since 1989. The hallmark of ILAC’s health portfolio is the *Cooperadores de Salud* (health co-operators) program, which trains volunteers to be CHWs leading health education and promotion efforts in the rural communities they reside in. The eight c*ooperadores* who were assigned to support CCI’s work at the two clinics were the target population of this study based on their lengthy careers as CHWs working with various projects and organizations. The group was chosen as the target population for this study because their long careers have allowed them to observe and experience changes over time, as the patterns of disease changed in their communities.

### Research team

The principal investigator for this study (CB) is a US-born public health researcher who has worked and lived in the DR for over 20 years, including collaboration with ILAC and the *cooperadores*. The interviewers, including the second author, were both of Latin American descent and completing graduate studies in public health at the time of the study. Both interviewers resided in one of the rural communities for the study duration. The first author is pursuing her doctoral degree in public health and is of Dominican origin, which facilitated both linguistic and cultural familiarity to aid in the analysis and interpretation of data.

### Data collection

All eight eligible *cooperadores* were invited to participate and all provided verbal informed consent. Interviewers read the informed consent form to the participants, and signed and dated the form confirming that the participant agreed to participate in the study. Because we were interested in their experiences as CHWs over a long span of time, we interviewed each *cooperador* twice to allow enough time to reflect on the past and present, and to follow up with clarifying questions, resulting in two interviews with each participant for a total of 16 interviews. All interviews were semi-structured, in-depth interviews between one interviewer and one participant and occurred from June to August.

The first round of interviews focused on *cooperadores*’ perceptions of changes in health concerns in their communities, how they believed these changes had occurred, and their role in these changes over the past 20 years. These interviews are the focus of this study. The second round of interviews focused on their current work in the diabetes and hypertension clinics and their perspectives on improving clinical care. We interviewed participants in private rooms in the clinics, their homes, and other community spaces such as schools. Interviews were conducted in Spanish, audio recorded, and lasted an average of 83 minutes (range 48–105 minutes). Audio recordings were transcribed verbatim by the two interviewers and two Dominican transcriptionists. All transcripts were reviewed against the audio for quality control. As all *cooperadores* in the study setting participated in the study, all transcripts were reviewed to remove identifying information to maintain participant confidentiality. This study received approval from the Institutional Review Board at the University of North Carolina (#15–1007). Permission to conduct the study at the participating clinics was granted by community leaders and our community partner organization, ILAC.

### Data analysis

During fieldwork, interviewers wrote field notes immediately following each interview and engaged in daily debriefing meetings to document initial impressions and observations of emerging themes. This process led to refining probes in subsequent interviews. Following fieldwork, EA read each transcript and wrote a narrative summary for each participant summarizing data focused on their perceptions of the health context and transitions in their communities, which were reviewed and discussed by all co-authors. We used the summaries to develop codes that captured how *cooperadores* viewed changes in their communities and roles over time. EA then systematically coded transcripts using ATLAS.ti 8. Some codes were descriptive, such as gastrointestinal disease and road conditions, while others were interpretive, such as gender dynamics and *cooperador* sustainability. After coding, EA summarized each code, integrated field notes, and combined similar codes to develop overarching themes. These themes were then developed through drafting the results in consultation with co-authors.

## Results

We interviewed eight *cooperadores* (n=7 women, n=1 man). Ages ranged from 47 to 65 years and education levels from 8th grade to university. Information about the participants is omitted in the results to protect anonymity. Many *cooperadores* were community members who were already leaders in their communities through church, mothers’, or women’s groups. *Cooperadores’* time working as CHWs ranged from 22 to 39 years with both governmental and non-governmental programs. They noted that their primary motivation for initiating and continuing their work as *cooperadores* was their commitment to helping the poor, which stemmed from their Christian beliefs.

The remaining results have been organized around the following themes: 1) health concerns of the past: a focus on maternal health and infectious disease; 2) health concerns of the present: double burden; 3) CHWs as advocates and healthcare navigators; 4) parallel transitions: the interacting trajectories of gender norms, educational attainment, and health; and lastly 5) sustainability.

### Health concerns of the past

*Cooperadores* identified that in the late 1990s and early 2000s, child morbidity and mortality was the most salient health concern in their communities. Most *cooperadores* began their health promotion work with a nutrition program for children under 5. They performed home visits to monitor weight, teach how to seek medical care for sick children, educate and support mothers on breastfeeding and proper feeding, and vaccinate children. When the ILAC program was established, *cooperadores’* work broadened to include prevention of infectious disease through education and improving access to personal and environmental hygiene, sanitation, clean water, and treatment for diarrheal disease (e.g., oral rehydration therapy). While the scope of work expanded, the target audiences remained the same: mothers and children under 5. *Cooperadores* expressed that these efforts led to increased knowledge about childhood disease transmission and management, and associated health behaviors. Participant 6 reflected on changes they observed:

The first accomplishment was changing people’s attitudes. Not everyone changed, but there was a beautiful and significant change, people learned to wash their hands [… and] drink boiled water, then [drink filtered water when] water filters came. -Participant 6

*Cooperadores* perceived that these improved health behaviors led to a drastic reduction in child morbidity and mortality, as described by Participant 8.

After we oriented mothers, everything began to change. From then to now, child mortality has disappeared because mothers listened to us… it’s not that diarrhea ended because you know diarrhea always exists, but compared with how it was before, diarrhea has disappeared. -Participant 8

Aside from primary prevention, *cooperadores* described how treatment knowledge and connections to care prevented child mortality: “After we had that development [having cooperadores], children stopped dying because now we know what to do [when they have distended stomachs from malnutrition or suffering from diarrhea].” (Participant 3) Although the primary roles described by *cooperadores* related to health education, they noted that the lack of access to adequate health infrastructure (e.g., latrines, clean water) impeded recommended health behaviors and increased exposure to disease-carrying vectors. Thus, in addition to educating, *cooperadores* advocated through ILAC and successfully improved community infrastructure by securing access to latrines, water filters, and improved access to running water. C*ooperadores’* work on adult health issues was limited to tuberculosis contact tracing, tetanus vaccination, and water safety education. They described not receiving much training about adult health in the early phases of their work.

### Present time: Double burden

In this section, we present how *cooperadores* described their evolving roles amidst the changing health needs in their communities. *Cooperadores* described how they currently respond to emerging adult health concerns, such as type 2 diabetes (T2D) and hypertension while still addressing lingering challenges related to infectious disease.

Their *cooperador* roles continued to include advocacy and education to prevent infectious outbreaks, and patient healthcare navigation. *Cooperadores* continued to advocate for improved public health infrastructure, including water, housing, and sanitation, as Participant 7 said: “We still need bathrooms. That has a lot to do with health and hygiene, houses need to have a bathroom.” Due to gaps in infrastructure, communicable diseases continued to be relevant even at the time of the study. For example, many referred to a recent dengue fever outbreak affecting their communities, as Participant 6 describes:

Now we have had a few cases of dengue, which is an alarm for us that our community is dirty, it is filling with plastics which fill with water in the streets and that is … the perfect condition to breed mosquitos. So this is a worry for us and we are taking up initiatives to see what we can do so there are no more [dengue] cases. -Participant 6

In response, some *cooperadores* provided community-wide health education on environmental hygiene, such as proper plastics disposal and removing standing water. Supplementary to community-based events, they used individualized approaches to address misconceptions or behaviors that may contribute to poor health outcomes and modeled recommended behaviors such as leading garbage clean-ups.

Further, NCDs, specifically T2D and hypertension, were most often noted as currently affecting their communities. The CCI program was initiated in 2010 to combat these conditions. Newly added roles were assisting with T2D and hypertension clinics (e.g., administrative, clinical, and patient education) and an expanded healthcare system navigation role, which will be discussed in detail in the next section. *Cooperadores* described continuing to be a community resource on health, specifically answering questions within and outside of the clinic on chronic disease management and how to seek care. *Cooperadores* tied increases in these chronic conditions to limited access to high-quality, nutritious foods, increased access to calorie-dense, processed foods, and limited dietary knowledge. *Cooperadores* believed the shift in access from ‘naturally grown’ foods to processed foods was ‘contaminating’ people’s bodies and affecting their overall health. Other health concerns in their communities included cancer, sexually transmitted diseases (e.g., HIV, HPV), drug use, kidney disease, problems with bone health, and generalized pain.

### The impact of cooperadores on healthcare access

Broadly, the *cooperador* role involves improving health-care access and helping community members navigate health-care systems. In the past, health navigation done by *cooperadores* focused on connecting children and families to healthcare in the context of referrals for extreme illness that could not be managed in the home. Over time, health navigation has taken over a greater proportion of their time with *cooperadores* accompanying patients to medical appointments in nearby cities. They also educate community members on how the healthcare system works (e.g., referrals, health insurance), and how to interpret post-visit instructions. Given the growing chronic disease burden, the patient population they serve is now broader, including men and people of all ages. The frequency of patient interactions increased from a few visits during pregnancy to providing continuous care over the life course to assist with chronic disease management.

*Cooperadores* also help facilitate *operativos*, when a group of external medical specialists sets up services to treat patients for free or a small quota. When the ILAC *cooperador* program began, *operativos* mainly consisted of eye exams and consultations, and then expanded to include OB/GYN, general consults, and dentistry. O*perativos* occur almost monthly, and *cooperadores* are responsible for informing, recruiting, and accompanying patients.

*Cooperadores* have always seen themselves as problem-solvers for their communities – from organizing trash clean-ups to advocating for a local preschool. One example of advocacy for improved healthcare access was their work to improve the roads in their communities. In the past, *cooperadores* described patients needing to travel 5–9 kilometers to the nearest healthcare facility on roads that were in such poor condition that motor vehicles could not cross. This meant that individuals often balanced their need for care with safety. Participant 7 recounted how this affected mortality, especially if someone got sick overnight:

Imagine that the city that was closest to us is [nearby city], and if someone gets sick at night, many times they would die because the journey was far. If they were seriously ill, they would die because the roads were very bad,... drivers did not want to leave at night because the roads were bad and because the city was far. -Participant 7

Participant 7 continued to recount that *cooperadores* advocated for better roads, targeting an important structural barrier to healthcare access.

Various groups would get together and organize… we would go on commissions to the authorities in [nearby city] to inform them that the roads have a lot of holes, a lot of dust, it was horrible. We went many, many times on commissions until one day, they listened to me. It’s like that, if we never went and never talked to them, [the roads] would have stayed the same forever. -Participant 7

Roads have now improved, and some communities have access to healthcare less than 1 kilometer away, with improved linkages to specialty and acute care facilities. More consistent and timely access helped reduce health complications and mortality. *Cooperadores* attributed this improved access in part to their advocacy, community outreach, and health education.

### Parallel transitions: Gender and education

The health transition co-occurred with a social transition in gender norms and roles. All *cooperadores* spoke about gender roles or dynamics either directly or indirectly. *Cooperadores* mentioned sexism as a health problem and social determinant of health, as reflected in the following quote. “[A health problem] 20 years ago that had a lot of influence was *machismo*; that men imposed [their will] on women and women had to be in the house dominated by men.” (Participant 1) Participant 1 continued explaining how gender roles led to childhood morbidities in the past due to how household resources were controlled.

We wanted the fathers to also attend workshops because they were the ones that mostly generated the resources. Not anymore,... but in those times... men went to get the resources, and women would just get what the men gave them. And they [the children] would suffer from malnutrition for that reason. Because men would do this in the household: “take this for you, and these are mine.” And he would take the majority for vices, and the women had to make do with little. It wasn’t enough even for them [much less for the kids]. -Participant 1

Participant 5 explained “it impacted me to see how men marginalized women in the community. Women were like a piece of furniture” so she set out to educate women about their rights. Women gathered to learn from her, and they eventually formed the Women’s Club. Participant 5’s effort to have women empower themselves and reframe gender norms pushed women across generations to pursue education, thus shifting the communities’ level of educational attainment.

When women began to realize that they had rights and responsibilities, they tried to improve themselves. They took courses and tried to change their families, in that not only men had the right to bring money home, but women also could...Women would then advise their daughters to keep studying, pursue a career... I remember that 20 years ago it wasn’t the same. Before perhaps every 1 or 2 houses had a person with a university degree. Now every house has 4 or 5.... I remember that before the teachers were from the cities; now the teachers are our neighbors. The nurses were from the city; now we are from the community.... There has been a great change. -Participant 5

As the gender transition expanded women’s possibilities to pursue education, it shaped the health transition as communities produced more health professionals. Participant 8 spoke about how her own experience attending the Women’s Club empowered her and prepared her to be able to undertake a *cooperador* role. Knowledge and training, particularly related to health, were also key to the empowerment of women. Participant 6 spoke about education as an asset that shaped the health transition:

What first motivated me was education. If I don’t prepare myself and study, if I don’t empower myself and make knowledge mine, then I can’t motivate others and I won’t have the motivation to work. I wanted my community to improve.

-Participant 6

Because the CHW role started mainly serving mothers and children, it began as a role exclusively performed by women. Despite the role broadening to serve all members of the community, most *cooperadores* continue to be women. *Cooperadores* theorized that as men traditionally worked outside the home, women were left as the only viable candidates to take on a largely volunteer position. The gendering of this role has led to even further asymmetrical responsibilities and burnout among women *cooperadores*. Women spoke about how difficult it was to balance the multiple roles of a *cooperadora* with the role of taking care of a family as a mother, wife, daughter, grandmother, and neighbor, as discussed by Participant 2:

What’s difficult for a cooperador? Well, the most limiting and difficult part for a cooperador is economic limitations. The economic part is limiting because not everyone is willing to let go of their responsibilities, especially women. Women have their husbands, their family, so while the husband is earning a living, when you come to look at it, the mom, the cooperadora must find an hour or two to leave the house or find a few days to [do the cooperador role]. -Participant 2

As women were empowered, they became leaders in their community and took initiative to mitigate or eliminate problems in their communities*.* However, the feminization of this role has implications for the sustainability of this model, as discussed below.

### Sustainability

*Cooperadores* expressed concerns with the sustainability of their work in two domains: workload and economics. When asked how their role was changing, Participant 6 said “Well, it’s changed because recently my job has grown.” They specified that their workload has increased because the scope of work has expanded in the clinic, for which they received a salary, while continuing to do health navigation and community outreach. One consequence of this expanded role within clinics was limiting other core aspects of their role, such as health education:

Interviewer: Are you doing health education workshops now in the community?

Participant 6: No, not right now. Currently no because we have had too much work here [in the clinic]. For example, in foot care, we have to examine patients’ feet... We’ve had numerous medical *operativos* in ILAC, and when that occurs, there are very few [cooperadores] that stay behind...

*Cooperadores* described difficulties balancing their many duties. Years into their health promotion career, Participant 2 reduced their workweek to 6 instead of 7 days a week to avoid burnout. Correspondingly, many c*ooperadores* described their role as constant, without a start or end to their schedule. Participant 4 said “I think I’m a cooperadora from the moment I wake up because my house became a community center. So, people always come to ask questions.” Participant 8 also spoke about individuals coming to their home for both informational and instrumental support, and described how the constant nature of the role interrupts their sleep and affects their well-being:

Sometimes I’m a bit unbalanced, because there’s a lot of things... there’s a lot of work daily and it’s a bit difficult sometimes when you go home and you’re tired, but life goes on and you have to continue no matter what… I’ve tried to give the most of myself when it comes to my work…[but] the most difficult part is that you’re home and you’re tired and want to rest but someone knocks on your door, whether it’s to give them medicine, to orient them, or to accompany a patient to the doctor.... [People apologize for waking me up] but I say, “Don’t worry, no problem, I’m here to serve.” -Participant 8

Despite their endless work and being champions in their communities, ILAC *cooperadores* are unpaid. The specific *cooperadores* we interviewed received a salary for their work supporting the CCI diabetes and hypertension clinics. A few also received a salary for their leadership role with the ILAC *cooperadores* program that went beyond the scope of their community work. Yet, as a whole, being a community health worker across different programs remains an unpaid position.

The voluntary nature of much of the work meant that many *cooperadores* did not have enough funds to secure adequate levels of food, clothes, and housing quality, the very same things they have continuously advocated for in their communities. Participant 1 explained, “Sometimes the cooperador…[has] to go [work] without even knowing if they are leaving a pot on [to cook a meal] in their house because there is no gas.” Financial precarity threatens the sustainability of the *cooperador* program. For instance, Participant 8 shared multiple accounts of being unable to accompany a patient to a health center because they didn’t have adequate clothes, food, or money for transportation for themselves. Although patients are supposed to pay their own and *cooperadores’* bus fares, many patients cannot afford their own fare, so *cooperadores* often fundraise or ask for personal loans to cover transportation costs to healthcare providers. *Cooperadores* noted that lack of payment is also a threat to retention:

We have trained many people to be cooperadores, but not a lot remain because the work does not provide for their needs. Being a cooperador is honorary, voluntary work. So many people have been trained but haven’t been able to maintain themselves in the community when they do the voluntary work, so they have left. - Participant 2

To address the double burden of infectious and NCDs, *cooperadores* are spread thinly across preventing infectious outbreaks, advocating for basic needs, addressing NCDs, being a community resource and health navigator, as well as meeting their own basic needs and handling gendered familial obligations.

## Discussion

We conducted interviews with *cooperadores* about how their communities’ health and their roles changed over the course of 20 years. *Cooperadores* described a transition in their communities from infectious diseases primarily affecting children to NCDs affecting a wider range of individuals. The *cooperador* role expanded to reflect both infectious and non-communicable health issues affecting these communities. *Cooperadores* considered themselves change agents who were instrumental in the health improvements in their communities. As they lived through the health transition, they also lived through a parallel social transition that saw the empowerment of women and increased pursuit of education in their communities, which supported improvements in health and directly affected the *cooperador* workforce.

While *cooperadores* described a transition, it was not linear or unidirectional from infectious to NCDs as previously theorized [12,13]. Instead, *cooperadores* described continued efforts to manage infectious diseases while addressing the emerging NCD burden – referred to as the double burden of disease [13]. McKeown and others have pointed to the limited generalizability of the epidemiologic transition framework to LMICs, especially for countries that continue to struggle with infectious epidemics [14]. While 65% of the burden of health in the DR is attributable to NCDs, 21% of disability-adjusted life years lost are still caused by nutritional, neonatal, maternal, and infectious diseases [16]. Between 2012 and 2018, the Dominican Republic experienced five disease outbreaks caused by the mosquito-borne viruses, specifically dengue, chikungunya, and Zika [19]. The COVID-19 pandemic is another reminder of how infectious diseases continue to pose a threat to public health and public health infrastructure globally.

Omran’s third proposition states that the most “profound changes in health and disease patterns [are] obtained among children and young women” during the epidemiological transition [12]. Our findings supported this proposition; *cooperadores* emphasized reduction in child morbidity and mortality as the most important health improvement they observed over time. Furthermore, the theoretical proposition and *cooperadores*’ perceptions of these declines are supported by epidemiological data. Between 1990 and 2022, under-5 child mortality almost halved in the Dominican Republic [20]. Yet, the country’s under-5 child mortality remains one of the highest in Latin America and the Caribbean, well above the regional average [20]. Several social determinants of health (SDOH) (e.g., water, sanitation) that cooperadores identified as the main drivers of child mortality and infectious disease in the past, were still issues in the present. These findings have been corroborated by other research studies in the country that have found a remaining issue of environmental sanitation with health impacts for both children and adults across vector-borne illnesses, and respiratory conditions [21].

These challenges are not limited to the Dominican Republic; rural populations in LMIC have significantly worse access to sanitation structures and clean water, compared with urban populations [22]. Omran’s revised theory suggests that for countries that do not experience improvements in SES and subsequent improvements in SDOH, infectious disease is reduced through public health programs instead, such as extensive vaccination campaigns [12]. Studies demonstrate CHW programs are one example of public health programming that has effectively reduced child morbidity and mortality in rural settings in low-income countries [23,24]. In our study, this was reflected in the participant narratives of health improvements achieved despite lingering inequities regarding SDOH, mainly through *cooperadores’* own efforts, such as life-saving oral rehydration education campaigns for children with dehydration and diarrheal disease and improved linkages to healthcare. Reflecting the prolonged-polarized model, the limited improvements in socioeconomic conditions and health infrastructures, including poor access to clean water and sanitation, limit the transition out of the infectious disease period not only for this community, but for many low-resourced, rural communities across the globe. Unlike the prolonged-polarized model, which predicted NCDs to be concentrated in urban areas, we found NCDs to be a heavy burden on the rural communities in this study and on the *cooperadores’* work.

C*ooperadores* described improving their communities’ health using a multilevel approach [25], including health education, community organizing, and advocacy to improve infrastructure and access. As members of the community, CHWs in this study and other settings are well positioned to understand and advocate for change in SDOH, from roads to oppressive gender dynamics, that affect preventive behaviors [26–28]. Advocacy is a core component of CHWs’ role that is effective at targeting underlying SDOHs and reducing health inequities, yet this key role is often pushed to the sidelines as CHWs are spread thin across other responsibilities [28–32]. Studies demonstrate that protected time for advocacy, specific advocacy and leadership training, and flexible work environments can facilitate this crucial component of their job that improves structural SDOH and leads to lasting change [29,33].

Further, the sustainability of *cooperadores'* work is threatened by three main factors: (1) high workload, (2) the unpaid nature of their role, and (3) gendered expectations within the home and community, all of which are linked. This is the first time CHW experiences and challenges have been qualitatively studied in DR and one of the first in LAC, but these same themes have also been found across CHW programs, such as in Kenya and India [18,34].

*Cooperadores* described an expansion of their scope of work, from focusing on infectious disease to now also managing rising NCD cases, particularly T2D and hypertension, as well as an expanded health navigation role. The double burden experienced in many LMICs coupled with under-resourced health systems have resulted in extended work for CHWs [18]. CHWs who have experienced an increased or excessive workload report being overwhelmed, demotivated, not having sufficient time for their domestic responsibilities, and having less time for other income-generating activities, with one study reporting that a third of their participants experienced loss of income as a result of being a CHW [34–40]. Aside from the effects of workload on individual wellbeing, *cooperadores* in this study also reported that their workload negatively impacted their ability to engage in health education. As roles expand or shift to disease-specific objectives or administrative tasks, programs are finding that CHWs have less time to connect with the community, engage in general health promotion, and target SDOH [3]. Addressing the excessive workload of CHWs as a result of their increased scope of work may require the assessment and revision of their current staffing policies, such as decreasing the catchment population per CHW [41].

Second, many *cooperadores* spoke about the impact of lack of payment on both their work and well-being, which is a common challenge among CHWs across the globe [40–45]. Globally, and specifically in Latin America, although some countries or programs provide CHWs with incentives, salaries, or short-term contracts; many CHW programs also largely depend on volunteers [3,4,37]. Yet, workload does not differ between paid and unpaid CHWs [18]. As noted earlier, CHWs in the DR who receive payment are generally paid below minimum wage [10]. This unpaid work rests “on the shoulders of impoverished women and men” [1,43]. As members of the target populations they serve, CHWs experience many of the same needs – with added unpaid work and work-related expenses [18].

Studies suggest that payment can empower CHWs, improve performance and motivation, and reduce attrition, especially when they have a high workload [42,45–47]. Participants in this study reflected both scenarios; while they struggled to meet their individual needs and the demands of their communities, they were highly dedicated to their volunteer and paid responsibilities. Studies suggest that like the cooperadores in this study, CHWs in many other settings often prioritize the needs of their communities above their own financial, emotional, and physical well-being [44,45,48–50].

Ministries of health and health funding agencies must consider the great value CHWs bring to community health and meet World Health Organization recommendation to incorporate a “financial package commensurate with job demands, complexity, number of hours worked, training and roles that they undertake” [41]. A salary will enable organizations to recruit new CHWs, support existing CHWs, and reduce attrition, which threatens the structure, sustainability, and cost-effectiveness of programs [51–53].

Beyond added workload and lack of payment, burnout is also exacerbated by the feminization of the CHW role, and the additional social burdens placed on women. Asymmetrical obligations – women being caregivers, men being breadwinners– worked together to produce a highly feminized CHW workforce, in this community and in other settings [54]. Being *cooperadoras* is added on to existing gendered caretaking roles and expectations, which in the current economic model, also includes contributing to household finances despite having limited earning potential [55,56]. *Cooperadores* describe the toll this takes, as they are physically tired of juggling multiple roles and anxious about neglecting their families financially and emotionally in their devotion to their community. Further, because of the financial and educational expectations on women produced with gender transitions, women may no longer be able to volunteer their time. These factors present a challenge to the current structure and sustainability of CHW programs.

Our study had limitations. First, when *cooperadores* reflected on their experiences, they did not always precisely reference years or points in time, so there is no further specificity than “before” and “current/after” to describe changes in their roles and communities. Second, while we aimed to identify private locations, given the open nature of space in the study setting, *cooperadores* may have felt uncomfortable openly discussing their experiences. Additionally, our study includes the experiences of a group of long-standing CHWs who are currently working in an NGO setting – which may or may not be different from the experiences of CHWs currently employed in government settings or those who have been working as CHWs for a short time.

Further research should explore the experiences of CHWs across Latin America and the Caribbean as the region’s health landscape continues to evolve. Community-based studies should be conducted to identify effective strategies for promoting the wellbeing of CHWs and the sustainability of CHWs' role in health education and advocacy for improved SDOH and health outcomes.

## Conclusions

As CHWs balance the dual burden of disease affecting many LMICs, programs need to protect core CHW functions such as advocacy and health education while expanding to include new services needed to support NCD care and management. CHW programs need to consider the impact of workload, remuneration, and gender roles on program sustainability, efficacy, and CHW well-being. Input from CHWs is critical for programming to generate sustainable and just models that challenge gender norms and empower CHWs and communities.

## Supporting information

S1 Checklist Inclusivity in global research. (DOCX)

## References

[1] Perry HB, Hodgins S. Health for the people: past, current, and future contributions of national community health worker programs to achieving global health goals. Glob Health Sci Pract. 2021;9(1):1–9. doi: 10.9745/GHSP-D-20-00459 33795359 PMC8087430

[2] Olaniran A, Smith H, Unkels R, Bar-Zeev S, van den Broek N. Who is a community health worker? - a systematic review of definitions. Glob Health Action. 2017;10(1):1272223. doi: 10.1080/16549716.2017.1272223 28222653 PMC5328349

[3] Zuñiga RO, Parra-García I, Gómez-Barrera LA. Overview of the participation of community health workers in primary health care in 6 Latin American countries and a proposal for their integration into the health system: a qualitative study. Fam Pract. 2024;41(2):139–46. doi: 10.1093/fampra/cmae002 38300797

[4] Méllo LMB de DE, Santos RCD, Albuquerque PC de. Community Health Workers: what do international studies tell us?. Cien Saude Colet. 2023;28(2):501–20. doi: 10.1590/1413-81232023282.12222022 36651403

[5] Ministerio de Salud Pública y Asistencia Social. Evaluación y Fortalecimiento de las Funciones Esenciales de Salud Pública en República Dominicana Periodo 2021- 2023 [Internet]. Santo Domingo, Reublica Dominicana: Ministerio de Salud Pública y Asistencia Social; 2023; p. 153–86. Available from: https://repositorio.msp.gob.do/bitstream/handle/123456789/2312/9789945644203%20%282%29.pdf?sequence=1&isAllowed=y

[6] República Dominicana. Reglamento General de Hospitales de la Republica Dominicana, No. 351-99. [Internet]. Consultoría Jurídica del Poder Ejecutivo, Ministerio de Salud Pública; 1999; p. 61. Report No.: 351–99. Available from: https://repositorio.msp.gob.do/handle/123456789/807

[7] Comisión Ejecutiva para la Reforma del Sector Salud (CERSS). Manual del promotor y la promotora de salud [Internet]. Vol. 4.3. Santo Domingo de Guzman, Reublica Dominicana, Secretaría de Estado de Salud Pública y Asistencia Social (SESPAS); 2009. Available from: http://repositorio.ministeriodesalud.gob.do//handle/123456789/1247

[8] Miller AS, Lin HC, Kang C-B, Loh LC. Health and Social Needs in Three Migrant Worker Communities around La Romana, Dominican Republic, and the Role of Volunteers: a thematic analysis and evaluation. J Trop Med. 2016;2016:4354063. doi: 10.1155/2016/4354063 27579046 PMC4989058

[9] El Nuevo Diario. El MAP, UTESA y el SNS gradúan 139 promotores de salud en Santiago y SD. El Nuevo Diario (República Dominicana) [Internet]. 2020 Jul 29; Available from: https://elnuevodiario.com.do/el-map-utesa-y-el-sns-graduan-139-promotores-de-salud-en-santiago-y-sd/

[10] Mejía M. Faltan 20 mil promotores de salud; nombrarán personal donde necesiten. Diario Libre [Internet]. 2012 Oct 8; Available from: https://www.diariolibre.com/actualidad/faltan-20-mil-promotores-de-salud-nombrarn-personal-donde-necesiten-JODL354841

[11] Bureau of democracy, human rights, and labor. Dominican Republic: 2009 Country Reports on Human Rights Practices [Internet]. U.S. Department of State; 2010. (Human Rights Report). Available from: //2009-2017.state.gov/j/drl/rls/hrrpt/2009/wha/136110.htm

[12] Omran AR. The epidemiologic transition: a theory of the epidemiology of population change. 1971. Milbank Q. 2005;83(4):731–57. doi: 10.1111/j.1468-0009.2005.00398.x 16279965 PMC2690264

[13] Boutayeb A. The double burden of communicable and non-communicable diseases in developing countries. Trans R Soc Trop Med Hyg. 2006;100(3):191–9. doi: 10.1016/j.trstmh.2005.07.021 16274715

[14] McKeown RE. The Epidemiologic transition: changing patterns of mortality and population dynamics. Am J Lifestyle Med. 2009;3(1 Suppl):19S-26S. doi: 10.1177/1559827609335350 20161566 PMC2805833

[15] Frenk J, Frejka T, Bobadilla JL, Stern C, Lozano R, Sepúlveda J, et al. The epidemiologic transition in Latin America. Bol Oficina Sanit Panam. 1991;111(6):485–96. 1838685

[16] Institute for Health Metrics and Evaluation (IHME). GBD compare data visualization. [Internet]. Seattle, WA: IHME: University of Washington; 2020. Available from: http://vizhub.healthdata.org/gbd-compare

[17] Country Profile of Dominican Republic [Internet]. Pan American Health Organization: World Health Organization; 2022 Sep. (Health in the Americas). Available from: https://hia.paho.org/en/countries-22/dominican-republic-country-profile

[18] Astale T, Abebe T, Mitike G. Workload and emerging challenges of community health workers in low- and middle-income countries: a mixed-methods systematic review. PLOS ONE. 2023;18(3):e10010520. 10.1371/journal.pone.0282717 PMC10010520 36913362

[19] Petrone ME, Earnest R, Lourenço J, Kraemer MUG, Paulino-Ramirez R, Grubaugh ND, et al. Asynchronicity of endemic and emerging mosquito-borne disease outbreaks in the Dominican Republic. Nat Commun. 2021;12(1):151. doi: 10.1038/s41467-020-20391-x 33420058 PMC7794562

[20] UN Inter-agency Group for Child Mortality Estimation. Levels & trends in child mortality [Internet]. 2023. Available from: https://data.unicef.org/resources/levels-and-trends-in-child-mortality/

[21] Turner C, Powell MA, Finalle RR, Westmoreland K, Osterhoudt K, Cordero Paulino R, et al. Talking trash: perspectives on community environmental health in the Dominican Republic. PLoS One. 2021;16(3):e0248843. doi: 10.1371/journal.pone.0248843 33780494 PMC8007031

[22] World Health Organization, United Nations Children’s Fund (UNICEF). Progress on sanitation and drinking water – 2015 update and MDG assessment [Internet]. Geneva: World Health Organization; 2015 [cited 2023 Mar 18]. Available from: https://apps.who.int/iris/handle/10665/177752

[23] Brenner JL, Kabakyenga J, Kyomuhangi T, Wotton KA, Pim C, Ntaro M, et al. Can volunteer community health workers decrease child morbidity and mortality in southwestern Uganda? An impact evaluation. PLoS One. 2011;6(12):e27997. doi: 10.1371/journal.pone.0027997 22194801 PMC3237430

[24] Perez F, Ba H, Dastagire SG, Altmann M. The role of community health workers in improving child health programmes in Mali. BMC Int Health Hum Rights. 2009;9:28. doi: 10.1186/1472-698X-9-28 19903349 PMC2782322

[25] Stokols D. Translating social ecological theory into guidelines for community health promotion. Am J Health Promot. 1996;10(4):282–98. doi: 10.4278/0890-1171-10.4.282 10159709

[26] Gurman T, Ballard Sara A, Villanueva Lorenzo F, Luis D, Hunter G, Maloney S, et al. The role of gender in Zika prevention behaviors in the Dominican Republic: findings and programmatic implications from a qualitative study. PLoS Negl Trop Dis. 2020;14(3):e0007994. doi: 10.1371/journal.pntd.0007994 32142512 PMC7112224

[27] Theobald S, MacPherson E, McCollum R, Tolhurst R. Close to community health providers post 2015: Realising their role in responsive health systems and addressing gendered social determinants of health. BMC Proceedings. 2015;9(10):S8. 28281706 10.1186/1753-6561-9-S10-S8 PMC4699124

[28] Nandi S, Schneider H. Addressing the social determinants of health: a case study from the Mitanin (community health worker) programme in India. Health Policy Planning. 2014;29(suppl_2):ii71-81. 25274643 10.1093/heapol/czu074 PMC4202921

[29] Ingram M, Sabo S, Rothers J, Wennerstrom A, de Zapien J. Community health workers and community advocacy: addressing health disparities. J Community Health. 2008;33(6):417–24. 18584315 10.1007/s10900-008-9111-y

[30] Ingram M, Schachter K, Sabo S, Reinschmidt K, Gomez S, De Zapien J. A community health worker intervention to address the social determinants of health through policy change. J Primary Prevent. 2014;35(2):119–23. 10.1007/s10935-013-0335-y PMC6742602 24363179

[31] Logan RI, Castañeda H. Addressing Health Disparities in the Rural United States: Advocacy as Caregiving among Community Health Workers and Promotores de Salud. Int J Environ Res Public Health. 2020;17(24):9223. doi: 10.3390/ijerph17249223 33321718 PMC7764816

[32] Rosenthal EL, Wiggins N, Ingram M, Mayfield-Johnson S, De Zapien JG. Community health workers then and now: an overview of national studies aimed at defining the field. J Ambul Care Manage. 2011;34(3):247–59. doi: 10.1097/JAC.0b013e31821c64d7 21673523

[33] Sabo S, Ingram M, Reinschmidt K, Schachter K, Jacobs L, de Zapien J. Predictors and a framework for fostering community advocacy as a community health worker core function to eliminate health disparities. Am J Public Health. 2013;103(7):E67–73. 10.2105/AJPH.2012.301108 PMC3682609 23678904

[34] Olaniran A, Madaj B, Bar-Zeev S, Banke-Thomas A, van den Broek N. Factors influencing motivation and job satisfaction of community health workers in Africa and Asia-A multi-country study. Int J Health Plann Manage. 2022;37(1):112–32. doi: 10.1002/hpm.3319 34476842

[35] Puett C, Coates J, Alderman H, Sadruddin S, Sadler K. Does greater workload lead to reduced quality of preventive and curative care among community health workers in Bangladesh?. Food Nutr Bull. 2012;33(4):273–87. doi: 10.1177/156482651203300408 23424894

[36] Condo J, Mugeni C, Naughton B, Hall K, Tuazon MA, Omwega A, et al. Rwanda’s evolving community health worker system: a qualitative assessment of client and provider perspectives. Hum Resour Health. 2014;12:71. doi: 10.1186/1478-4491-12-71 25495237 PMC4320528

[37] Scott K, Beckham SW, Gross M, Pariyo G, Rao KD, Cometto G, et al. What do we know about community-based health worker programs? A systematic review of existing reviews on community health workers. Hum Resour Health. 2018;16(1):39. doi: 10.1186/s12960-018-0304-x 30115074 PMC6097220

[38] Kasteng F, Settumba S, Källander K, Vassall A, inSCALE Study Group. Valuing the work of unpaid community health workers and exploring the incentives to volunteering in rural Africa. Health Policy Plan. 2016;31(2):205–16. doi: 10.1093/heapol/czv042 26001813 PMC4748129

[39] Javanparast S, Baum F, Labonte R, Sanders D. Community health workers’ perspectives on their contribution to rural health and well-being in Iran. Am J Public Health. 2011;101(12):2287–92. doi: 10.2105/AJPH.2011.300355 22021303 PMC3222427

[40] John A, Nisbett N, Barnett I, Avula R, Menon P. Factors influencing the performance of community health workers: a qualitative study of Anganwadi Workers from Bihar, India. PLoS One. 2020;15(11):e0242460. doi: 10.1371/journal.pone.0242460 33237939 PMC7688170

[41] WHO guideline on health policy and system support to optimize community health worker programmes. Geneva: World Health Organization; 2018. https://iris.who.int/handle/10665/275474 30431747

[42] Closser S, Napier H, Maes K, Abesha R, Gebremariam H, Backe G, et al. Does volunteer community health work empower women? Evidence from Ethiopia’s Women’s Development Army. Health Policy Plan. 2019;34(4):298–306. doi: 10.1093/heapol/czz025 31143930

[43] Maes K, Closser S, Kalofonos I. Listening to community health workers: how ethnographic research can inform positive relationships among community health workers, health institutions, and communities. Am J Public Health. 2014;104(5):e5-9. doi: 10.2105/AJPH.2014.301907 24625167 PMC3987580

[44] Maes K, Closser S, Tesfaye Y, Gilbert Y, Abesha R. Volunteers in Ethiopia’s women’s development army are more deprived and distressed than their neighbors: cross-sectional survey data from rural Ethiopia. BMC Public Health. 2018;18(1):258. doi: 10.1186/s12889-018-5159-5 29444660 PMC5813408

[45] Topp SM, Price JE, Nanyangwe-Moyo T, Mulenga DM, Dennis ML, Ngunga MM. Motivations for entering and remaining in volunteer service: findings from a mixed-method survey among HIV caregivers in Zambia. Hum Resour Health. 2015;13:72. doi: 10.1186/s12960-015-0062-y 26329324 PMC4557603

[46] Kok MC, Dieleman M, Taegtmeyer M, Broerse JEW, Kane SS, Ormel H, et al. Which intervention design factors influence performance of community health workers in low- and middle-income countries? A systematic review. Health Policy Plan. 2015;30(9):1207–27. doi: 10.1093/heapol/czu126 25500559 PMC4597042

[47] Alam K, Tasneem S, Oliveras E. Retention of female volunteer community health workers in Dhaka urban slums: a case-control study. Health Policy Plan. 2012;27(6):477–86. doi: 10.1093/heapol/czr059 21900361

[48] Busza J, Dauya E, Makamba M, Ferrand RA. “I will not stop visiting!” a qualitative study of community health workers’ reluctance to withdraw household support following the end of a community-based intervention in Zimbabwe. BMC Health Serv Res. 2018;18(1):718. doi: 10.1186/s12913-018-3531-x 30223831 PMC6142685

[49] Maes K, Kalofonos I. Becoming and remaining community health workers: perspectives from Ethiopia and Mozambique. Soc Sci Med. 2013;87:52–9. doi: 10.1016/j.socscimed.2013.03.026 23631778 PMC3732583

[50] Pandey J, Singh M. Donning the mask: effects of emotional labour strategies on burnout and job satisfaction in community healthcare. Health Policy Plan. 2016;31(5):551–62. doi: 10.1093/heapol/czv102 26491059

[51] Alam K, Khan JAM, Walker DG. Impact of dropout of female volunteer community health workers: an exploration in Dhaka urban slums. BMC Health Serv Res. 2012;12:260. doi: 10.1186/1472-6963-12-260 22897922 PMC3464156

[52] Rahman SM, Ali NA, Jennings L, Seraji MHR, Mannan I, Shah R, et al. Factors affecting recruitment and retention of community health workers in a newborn care intervention in Bangladesh. Hum Resour Health. 2010;8:12. doi: 10.1186/1478-4491-8-12 20438642 PMC2875202

[53] Walt G, Perera M, Heggenhougen K. Are large-scale volunteer community health worker programmes feasible? The case of Sri Lanka. Social Sci Med. 1989;29(5):599–608. 10.1016/0277-9536(89)90179-2 2799410

[54] Villa-Torres L, Fleming PJ, Barrington C. Engaging men as promotores de salud: perceptions of community health workers among Latino men in North Carolina. J Community Health. 2015;40(1):167–74. doi: 10.1007/s10900-014-9915-x 24989349 PMC4710487

[55] Safa HI. The myth of the male breadwinner: women and industrialization in the Caribbean. Boulder: Westview Press; 1995. 208 p. (Conflict and social change series).

[56] Steege R, Taegtmeyer M, McCollum R, Hawkins K, Ormel H, Kok M, et al. How do gender relations affect the working lives of close to community health service providers? Empirical research, a review and conceptual framework. Soc Sci Med. 2018;209:1–13. doi: 10.1016/j.socscimed.2018.05.002 29777956

